# Comparison between different radiographic methods for evaluating the flexibility of scoliosis curves

**DOI:** 10.1590/1413-78522014220200844

**Published:** 2014

**Authors:** Luciano Miller Reis Rodrigues, Fabrício Hitoshi Ueno, Alberto Ofenhejm Gotfryd, Thiago Mattar, Edison Noboru Fujiki, Carlo Milani

**Affiliations:** Faculdade de Medicina do ABC, Santo André, SP, Brazil, Spinal Column Surgery Group, Faculdade de Medicina do ABC - Santo André, SP, Brazil

**Keywords:** Scoliosis, Radiography, Preoperative care

## Abstract

**OBJECTIVE:**

To compare different radiographic methods of spine evaluation to estimate the reducibility and flexibility of the scoliosis curves.

**METHODS:**

Twenty one patients with Lenke types I and III adolescent idiopathic scoliosis (AIS) were included. Radiographic evaluations were made preoperatively on the orthostatic, supine decubitus with lateral inclination to the right and left and supine positions with manual reduction, with support in the apex of each curve on the X-ray table. On the day of surgery, when the patient was anesthetized, radiography was taken with longitudinal traction through divergent forces, holding under the arms and ankles, and with translational force at the apex of the deformity for curve correction. After one week, a post-operative radiography was performed in orthostatic position.

**RESULTS:**

The correction and flexibility of the main thoracic and thoracic/lumbar curves were statistically different between the supine radiographs, manual reduction, modified traction under general anesthesia, lateral inclination and postoperatively. The modified maneuver for traction under general anesthesia is the one which showed greater flexibility, besides presenting higher radiographic similarity to postoperative aspects.

**CONCLUSION:**

Among the radiographic modalities evaluated the study under anesthesia with traction and reduction showed better correlation with postoperative radiographic appearance.*** Level of Evidence IV, Case Series. ***

## INTRODUCTION

Surgical correction of scoliosis is aimed at obtaining coronal, sagittal and axial balance of the spine with the lowest fused levels.^1^ ^,^ ^2^ The advantages of selective arthrodesis are: less blood loss, preservation of movable segments and decreased risk of pseudoartrose.^3^ To this end, the preoperative analysis of the structure of curves and evaluation of its flexibility is a key part in surgical planning.^4^

The preoperative flexibility of the curves can be evaluated using radiographs in lateral tilt when standing upright or supine radiographic study under traction.^5^ ^,^ ^6^ Radiographs in supine position with maximum slopes for preoperative evaluation have been performed most commonly, since they are inexpensive and easy to interpret.^7^ ^,^ ^8^ However, several other methods are also used, such as gradients in the standing position, longitudinal traction in supine position, tilt with fulcrum at the apex of the curve and sedated radiographic study.

A standardized assessment of curve flexibility in patients with adolescent idiopathic scoliosis (AIS), preoperatively, allows better comparison of results from different studies, besides being a useful tool in surgical planning.^9^ Therefore, the aim of this study is to evaluate the flexibility of scoliotic curves in patients with AIS by standard radiographs in supine and lateral tilt preoperatively and after anesthesia with manual correction, as well as its correlation with post- surgical corrective results.

## MATERIALS AND METHODS

A convenience sample of 21 patients with AIS structured curves was studied according to the criteria of Lenke *et al.* ^10^ treated surgically with posterior instrumentation with pedicle screws^11^. Three male and 18 female patients were enrolled in this study, aged between 13 and 19 years old (mean age 15 years and 3 months old).

The following inclusion criteria were used: patients with Lenke curve types I and III. Exclusion criteria were: Lenke curves types 2, 4, 5 and 6; patients who underwent reoperation, need for thoracoplasty, osteotomy and anterior approach. Patients with thoracic curve to the left were also excluded.

All patients underwent surgical correction of scoliosis and instrumentation by posterior approach. In all cases, only monoaxial pedicle screws were used and no hooks or sublaminar strings were used in the assembly. Density of the implants (ratio between the number of implants used in the assembly and the total number of sites available for implants) was assessed, as recommended by Suk *et al*.^12^ The method used for curve correction maneuver was "derotation" of the rod in the concavity, as described by Cotrel *et al*.^13^

Before surgery, each patient underwent the first radiograph in the standing position and the second in supine decubitus with right and left leaning. The third radiograph was performed in supine position with manual reduction, with support in the summits of each curve in the X-ray table, as described by Kleinman *et al*.^14^ All inclination radiographs and manual reduction were supervised by a spine surgeon during ambulatory follow up.

On the day of surgery, with the patient under anesthesia, the fourth radiograph was performed, immediately before surgery, with the patient in supine position. For this radiograph longitudinal traction by divergent strength under armpits and ankles held by two spine surgeons. Translational force at the apex of the thoracic deformity was also held, for correction of the curve.

One week after scoliosis correction surgery, the fifth radiographic study, in orthostatic position, was performed.

In all analyzed radiographs, the Cobb angle was measured for primary thoracolumbar curve and thoracic curve. The lower limit for main thoracic curve was the apex above or equal to T11/T12 disc.

Data was organized and tabulated, then subjected to statistical analysis to calculate significance. A level of significance of 5 % (p<0.050) was adopted. The Statistical Package for Social Sciences (SPSS) program was used in its version 19.0, to obtain the results.

## RESULTS

Tables 1 and 2 show flexibility and correction of main thoracic curve (T MAIN) and thoracic-lumbar/lumbar curve (TL/L).

**Table 1:** Mean flexibility of main thoracic curves, standard deviation, and statistical difference between these variables (Friedman's test).

Variables	n	Mean	Standard deviation	Min	Max	Percentile 25	Percentile 50 (Median)	Percentile 75	p
Main thoracic supine	15	67,33	18,17	42,00	105,00	55,00	61,00	82,00	< 0,001
Main manual thoracic reduction	15	52,73	23,70	18,00	98,00	35,00	49,00	76,00	
Main modified thoracic traction	15	39,93	20,72	16,00	82,00	26,00	30,00	60,00	
Main thoracic Inclination	15	54,33	21,98	25,00	100,00	38,00	54,00	77,00	
Main thoracic post-operative	15	42,80	18,41	22,00	75,00	27,00	35,00	60,00	

**Table 2:** Mean flexibility of thoracic-lumbar and lumbar curves, standard deviation, and statistical difference between these variables (Friedman's test).

Variables	n	Mean	Standard deviation	Min	Max	Percentile 25	Percentile 50 (Median)	Percentile 75	p
Supine thoracic lumbar/lumbar	12	50,83	14,24	32,00	90,00	42,25	49,50	54,50	< 0,001
Manual thoracic lumbar/lumbar reduction	12	31,00	15,78	5,00	68,00	25,25	28,50	37,75	
Modified thoracic lumbar/lumbar traction	12	27,25	15,67	6,00	60,00	13,50	25,50	34,25	
Thoracic lumbar/lumbar Inclination	12	37,50	17,13	4,00	75,00	31,25	37,00	43,00	
Thoracic lumbar/lumbar post-operative	12	34,75	16,25	12,00	75,00	26,00	30,00	42,50	

Table 1 shows that there was no statistical difference between the supine radiographs, manual reduction (RED MAN), modified traction under general anesthesia (TRA MOD), lateral tilt and postoperative (PO) in the main thoracic curves, as in Table 2 with the thoracic-lumbar/lumbar curve (TL /L). The modified traction maneuver under general anesthesia showed greater flexibility and reducibility of the main thoracic curves and also in thoracic-lumbar/lumbar curve (TL/L), besides resembling the postoperative radiographic study. Table 3 shows the results of *post-hoc* analysis with Wilcoxon signed-rank test, comparing the five variables pairwise tended to show differences.

**Table 3:** Comparison between variables, pairwise through Wilcoxon signed-rank test, adjusted by Bonferroni correction*.

Variables pair	p
Main manual thoracic reduction - Main thoracic supine	0,018
Main modified thoracic traction - Main thoracic supine	0,008
Main thoracic Inclination - Main thoracic supine	0,011
Main thoracic post-operative - Main thoracic supine	0,008
Main modified thoracic traction - Main manual thoracic reduction	0,008
Main thoracic Inclination – Main manual thoracic reduction	0,440
Main thoracic post-operative – Main manual thoracic reduction	0,008
Main thoracic Inclination - Main modified thoracic traction	0,008
Main thoracic post-operative – Main modified thoracic traction	0,399
Main thoracic post-operative - Main thoracic Inclination	0,012
Manual thoracic lumbar/lumbar reduction - thoracic lumbar/lumbar supine	0,008
Modified thoracic lumbar/lumbar traction - thoracic lumbar/lumbar	0,008
Thoracic lumbar/lumbar Inclination - thoracic lumbar/lumbar supine	0,008
Thoracic lumbar/lumbar post-operative - thoracic lumbar/lumbar supine	0,008
Modified thoracic lumbar/lumbar traction – Manual thoracic lumbar/lumbar reduction	0,123
Thoracic lumbar/lumbar Inclination – Manual thoracic lumbar/lumbar reduction	0,008
Thoracic lumbar/lumbar post-operative – Manual thoracic lumbar/lumbar reduction	0,011
Thoracic lumbar/lumbar Inclination – Manual thoracic lumbar/lumbar traction	0,007
Thoracic lumbar/lumbar post-operative – Manual thoracic lumbar/lumbar traction	0,013
Thoracic lumbar/lumbar post-operative - Thoracic lumbar/lumbar Inclination	0,027

Regarding the main moderate thoracic curves (≤ 65 degrees), proportionally we found no statistically significant difference between the radiographs during the reduction maneuver. The same was observed in severe curves (65 degrees) as shown in Table 4. No statistically significant difference in thoracic-lumbar/lumbar curves was found in both serious as in moderate difference, as shown in Table 5.

**Table 4:** Differences between reduction maneuvers of main thoracic curves with Cobb angles lower and higher than 65 degrees simultaneously compared through Cochran test.

Variables	≤ 65		> 65		p
	Frequency	Percentage	Frequency	Percentage	
Main thoracic supine	11	68,80%	5	31,30%	0,115
Main manual thoracic reduction	12	75,00%	4	25,00%	
Main modified thoracic traction	15	88,20%	2	11,80%	
Main thoracic Inclination	11	73,30%	4	26,70%	
Main thoracic post-operative	13	81,30%	3	18,80%	

**Table 5:** Differences between reduction maneuvers of thoracic lumbar/ lumbar curves with Cobb angles lower and higher than 65 degrees simultaneously compared through Cochran test.

Variables	≤ 65		> 65		p
	Frequency	Percentage	Frequency	Percentage	
Thoracic lumbar/lumbar supine	14	93,30%	1	6,70%	0,406
Manual thoracic lumbar/lumbar reduction	14	93,30%	1	6,70%	
Modified thoracic lumbar/lumbar traction	14	100,00%	0	0,00%	
Thoracic lumbar/lumbar Inclination	15	93,80%	1	6,30%	
Thoracic lumbar/lumbar post-operative	12	92,30%	1	7,70%	

## DISCUSSION

Flexibility radiographs have been recommended to help determine the surgical technique and the levels to be selected in the correction of scoliosis. Lateral bending radiographs were described for the accuracy of the surgical correction with first generation instruments (Harrington system) and are considered the gold standard for this purpose. However, with the use of pedicle screws, the predictive value of active side slope supine radiographs began to be questioned. Gotfryd *et al*.^7^demonstrated that the method is predictive of the correction achieved exclusively with pedicle screws. However, in some situations, it is difficult to obtain cooperation from patients to perform the exam, a fact that complicates the evaluation of flexibility of curves.^15^ ^,^ ^16^ A false interpretation of the stiffness of a curve may induce the surgeon to make unnecessary fusions. Thus, other methods of evaluation of the curve flexibility have been proposed.

Various types of radiographs have been advocated to assess the flexibility of the curves, such as manual reduction on the apex of curve.^14^ Cheung and Luk^17^ evaluated flexibility through radiographs with slope associated with corrective force, with the fulcrum at the apex of the curve, and compared these results with those obtained on radiographs with slope and postoperative correction achieved in thoracic curves after posterior fusion. However, the authors found no significant difference.^17^ ^,^ ^18^ Davis *et al.* ^19^described the radiographic technique traction under general anesthesia in the operating environment, which, according to the author, best resemble the radiographic appearance after corrective surgery. In our study, we used three types of deformity correction techniques to evaluate the flexibility of the curve: X-ray tilt supine radiographs with manual reduction of the curve at the apex of the deformity and radiography with traction after anesthesia.

In this study, we performed radiography with manual reduction as described by Kleinman *et al.* ^14^ and observed no significant difference compared to other correction methods. However, when analyzed separately, the location of the curve, no significant difference for both main thoracic curves and for the thoracic-lumbar/lumbar was observed. This led us to suggest that the efficacy of the test was not affected by the location of the curve. Similarly, Kleinman *et al*.^14^ observed that the effectiveness of manual reduction was also not altered by the location of the curve, pattern (single or double curve) or scoliosis etiology.

Regarding supine radiographs, we believe that the main drawback is the difficulty to standardize the force to be exerted during correction, besides the degree of relaxation of the patient during the examination, factors that directly affect the degree of correction of the deformidade.^3^ Manual reduction in pronation is still useful in predicting the behavior of the curves at the levels that will not be submitted to arthrodesis. 

Traditionally, in traction radiographs are performed in patients less capable of performing lateral inclination (not collaborative patient or in case of neuromuscular scoliosis).^1^ Traction under general anesthesia is a relatively new technique, first reported by Davis *et al*.^19^ The flexibility of curve in traction under general anesthesia is enhanced by muscle relaxation of the patient, there is no discomfort and does not required the patient's collaboration.^17^ Through it, one can more easily standardize the degree of force being exerted by the examinator.^4^ In present study, it was found that the tensile radiographs show greater similarity with surgical correction against the main thoracic curves. Regarding lumbar curves, the reducibility was larger than the correction achieved postoperatively, which may be explained by the very muscular relaxation obtained with the method. To our knowledge, no previous study had described this phenomenon.

Regarding severe curves (greater than 65 Cobb degrees) no statistical difference was noted in traction under general anesthesia for both main thoracic curves as in thoracic-lumbar/lumbar curves. Radiography with traction under general anesthesia showed greater flexibility when compared to supine lateral tilt and tilt with fulcrum at the apex of the curve for high degree curves (Cobb angle>65 degrees) and rigid ones; however, this result was not statistically significant, possibly due the small sample of patients with curves of high degree. In a more recent study, traction under general anesthesia was compared to the same side slope in the supine and thoracic-lumbar/lumbar main thoracic curves and, when divided into severe curves (65 degrees) and moderate, they were again equivalent, with a tendency to greater flexibility for traction under general anesthesia in main severe thoracic curves.^20^

A limiting factor for potential clinical application of this type of radiography is that the surgeon cannot give the patient a preoperative planning set before general anesthesia geral.^4^ It is also difficult to obtain quality radiographs in the operating room for evaluation and measuring curves. In the present study, the sample was selected and a recruitment bias may be present.

## CONCLUSION

It was shown that the radiographic postoperative appearance in Lenke's type I and III adolescent idiopathic scoliosis, treated with a posterior spinal fusion, can be better predicted from analysis of radiographic imaging under general anesthesia with traction and manual reduction.
